# Respiration-entrained brain oscillations in healthy fMRI participants with high anxiety

**DOI:** 10.1038/s41598-023-29482-3

**Published:** 2023-02-10

**Authors:** Gert Pfurtscheller, Maciej Kaminski, Katarzyna J.Blinowska, Beate Rassler, Gerhard Schwarz, Wolfgang Klimesch

**Affiliations:** 1 Institute of Neural Engineering, Graz University of Technology, Graz, Austria; 2 Faculty of Physics, University of Warsaw, ul. Pasteura 5, 02-093 Warsaw, Poland; 3 Nalecz Institute of Biocybernetics and Biomedical Engineering, Polish Academy of Sciences, Ks. Trojdena 4 St., 02-109 Warsaw, Poland; 4 Carl-Ludwig-Institute of Physiology, University of Leipzig, Leipzig, Germany; 5 Department of Anaesthesiology and Intensive Care Medicine, Medical University of Graz, Graz, Austria; 6 Centre of Cognitive Neuroscience, University of Salzburg, Salzburg, Austria

**Keywords:** Dynamical systems, Respiratory system models

## Abstract

Brain-body interactions can be studied by using directed coupling measurements of fMRI oscillations in the low (0.1–0.2 Hz) and high frequency bands (HF; 0.2–0.4 Hz). Recently, a preponderance of oscillations in the information flow between the brainstem and the prefrontal cortex at around 0.15/0.16 Hz was shown. The goal of this study was to investigate the information flow between BOLD-, respiratory-, and heart beat-to-beat interval (RRI) signals in the HF band in healthy subjects with high anxiety during fMRI examinations. A multivariate autoregressive model was concurrently applied to the BOLD signals from the middle frontal gyrus (MFG), precentral gyrus and the brainstem, as well as to respiratory and RRI signals. Causal coupling between all signals was determined using the Directed Transfer Function (DTF). We found a salience of fast respiratory waves with a period of 3.1 s (corresponding to ~ 0.32 Hz) and a highly significant (*p* < 0.001) top-down information-flow from BOLD oscillations in the MFG to the brainstem. Additionally, there was a significant (*p* < 0.01) information flow from RRI to respiratory oscillations. We speculate that brain oscillations around 0.32 Hz, triggered by nasal breathing, are projected downwards to the brainstem. Particularly interesting is the driving force of cardiac to respiratory waves with a ratio of 1:1 or 1:2. These results support the binary hierarchy model with preferred respiratory frequencies at 0.32 Hz and 0.16 Hz.

## Introduction

The observation of the driving force of nasal respiration on neural oscillations is known since many years^1^. Although many studies on the rodent brain have documented oscillations in the olfactory bulb induced by nasal breathing^2–5^, data from humans are lacking. These respiration-entrained rhythms in animals are global, but often overlooked, although they are dominant in the delta and theta frequency range due to the high respiration rate. Studies in patients with medically intractable epilepsy and slower respiration rhythms of ~ 0.16 to 0.32 Hz revealed nasal respiration-entrained limbic oscillations in the piriform (olfactory) cortex, as well as in the amygdala and hippocampus^6,7^. In contrast to rodents, in humans the spatial organization of the glomerular-layer and odor-evoked potentials are different^8^.

Studies on nasal versus oral breathing in humans are rare, but revealed interesting functional connectivity (FC) differences. While mouth breathing is more left lateralized, nasal breathing had a more symmetrical FC pattern^9^. Sensorimotor areas were most important in both types of breathing, while brainstem regions appeared more often activated with nasal breathing. A cognitive study (two-back working memory task) showed stronger brain activation in fMRI recordings and connectivity during nasal breathing than during oral breathing^10^. Especially, the left cerebellum, left and right inferior parietal gyrus are activated in relationship to working memory. This is significant evidence that oral breathing is inappropriate, when using brain imaging techniques in cognitive studies. One problem is the exact control of both breathing types. Nasal clips can prevent respiration via nose, but oral breathing cannot be prevented completely during nasal breathing.

The findings of Zelano et al.^6^ obtained with intracranial EEG recordings in patients and nasal respiration-entrained oscillations of ~ 0.16 to 0.32 Hz are of special interest for the following reasons: (i) frequencies of 0.16 Hz and 0.32 Hz are preferred breathing frequencies in the binary hierarchy model of Klimesch^11^, (ii) the period duration (PD) of a breathing wave of ~ 3.1 s (which corresponds to oscillations at ~ 0.32 Hz) dominates during elevated anxiety in healthy fMRI participants^12^ and (iii) oscillations around 0.16 Hz are very likely associated with pacemaker-like activity in the brainstem prevail in states of anxiety^13^. Therefore, the question arises, whether there is a relationship between respiration-entrained oscillations at ~ 0.16 Hz and ~ 0.32 Hz and anxiety processes in the limbic system.

The bi-directional information flow between respiration, cortex and brainstem can be studied directly in animal experiments, in patients with intracranial electrode arrays, and via BOLD signal recordings in normal human subjects. The calculation of the Directed Transfer Function (DTF) based on the Granger causality principle offers a way to investigate directed coupling between different physiological signals^14,15^. The base for DTF is a multivariate autoregressive (MVAR) model, which is fitted simultaneously to the physiological signals including BOLD recording from prefrontal cortex and brainstem. This method of processing was already successfully applied to cardiac beat-to-beat interval (RRI) time courses, respiration and BOLD data in the low frequency (LF) bands of 0.05–0.15 Hz and 0.1–0.2 Hz^16,17^.

The goal of this paper is to study the information flow between the prefrontal cortex (PFC), brainstem, respiration, and RRI signals in the HF band (0.2–0.4 Hz) in a group of ten selected healthy fMRI participants with high anxiety (Rassler et al.^12^). Important questions are: (i) Do the results of DTF calculations in the HF band allow any conclusions about nasal respiration and (ii) can respiratory oscillations at ~ 0.3 Hz be induced through slow RRI fluctuation?

## Methods

### Study approval

All participants gave informed written consent to the protocol of the study, which was approved by the local Ethics Committee at the University of Graz (number: GZ. 39/75/63 ex 2013/14). Our research was performed in accordance with the ethical standards laid down in the 1964 Declaration of Helsinki.

### Goal of the Study

In a group of ten healthy fMRI participants with high anxiety score the directed (causal) coupling was studied between BOLD, respiration and cardiac beat-to-beat interval (RRI) oscillations in the high frequency band 0.2–0.4 Hz.

### Experimental paradigm

The experimental design consisted of four resting states (R1, R2, R3, R4) each lasting for 350 s. In each resting state the state anxiety (AS) was assessed with the state-trait anxiety and depression inventory (STADI^18^). Respiration and the electrocardiogram (ECG) were recorded inside the scanner. Details about the exact timing see^16,17^.

### Participants

A total of N = 23 participants (12 female, 22 right-handed) between 19 and 34 years of age (M = 24, SD = 3.2 years) took part in the fMRI study. Participants were naïve to the purpose of the study, had no former MRI experience and were without any record of neurological or psychiatric disorders (assessed by self-report). From these 23 participants with fMRI-related anxiety already two publications are ble in Scientific Reports^16,17^ which focused on the two low frequency bands of 0.05–0.15 Hz and 0.1–0.2 Hz. In this paper we studied the high frequency band 0.2–0.4 Hz in a group of ten fMRI participants with the highest anxiety scores (AS). AS ranged from 10 (no anxiety) to 40 (highest) with AS = 24.6 +  − 2.5. These ten participants have been subjected to an extensive wave-by-wave analyses^12^.

### Physiological signal recording and RRI time courses

ECG from thorax and respiration with a chest belt were recorded. The sampling rate was 400 Hz. QRS detection and subsequent computation of RRI time series were performed using fMRI plug-in for EEGLAB^19^. For further improvement of cardiac RRI detection the Kubios HRV Premium Package^20^ was used. For details of the wave-by-wave analyses of breathing and RRI waves see^12^.

### Resting state fMRI and ROI selection

Functional images were acquired with a 3 T scanner (Magneton Skyra, Siemens, Erlangen, Germany) using a multiband GE-EPI sequence^21^ with a simultaneous six-band acquisition with TE/TR = 34/871 ms, 52° flip angle, 2 × 2 × 2 mm^3^ voxel size, 66 contiguous axial slices (11 × 6), acquisition matrix of 90 × 104 and a FOV of 180 × 208 mm^2^. Finally, the AAL atlas^22^ was used to extract time courses for specified regions of interest (ROIs) in the left pre-central gyrus (PCG, ROI 1), left middle frontal gyrus (MFG, ROI 7), left insula (ROI 29), left amygdala (ROI 41), left hippocampus (ROI 37), left frontal medial orbital part (ROI 25), left brainstem (ROI 93, ROI 103) and right brainstem (ROIs 96, 98, 100).

### Computing of causal coupling and statistics

The interaction between the time series was studied by means of DTF^14^, which allows to estimate causal coupling between signals as a function of frequency. DTF is based on the Granger causality principle^23^, which gives a statement on predictability for two time series. If the variance of the prediction error for the second time series is reduced by including past measurements from the first time series in the linear regression model, then the first time series can be said to cause the second time series. Granger causality principle is equivalent to the 2-channel Multivariate Autoregressive Model (MVAR) but it may be extended to an arbitrary number of channels. For this study MVAR was extended to 6 channels^15^. Epochs of 40 s were used for the analysis, and the bootstrap approach was employed for the statistics. More details can may be found in^16,17^.

## Results

The group of 10 fMRI participants with high anxiety scores (AS = 24.6 ± 2.5) has been subjected to a wave-by-wave analysis, where the PDs of single breathing and cardiac RRI waves were determined^12^. The results of this wave-by-wave study are summarized in Table 1. The healthy subjects with high anxiety displayed a large number of slow RRI waves with PD = 10.2 s ± 0.64 (corresponds to ~ 0.1 Hz oscillations) and PD = 6.7 s ± 0.21 (corresponds to ~ 0.15 Hz oscillations) and also a large number of breathing waves with PD = 3.2 s ± 0.26 (corresponds a respiration frequency of ~ 0.31 Hz). While the RRI waves with PD of 6.7 s appeared in about 24% of the resting state duration of 320 s, the fast breathing waves appeared in about 71%.

**Table 1:** A summary of the results of the wave-by-wave analyses from 10 fMRI participants with high anxiety (modified from^12^).

Subject	AS	BOLD analysis			RRI				Resp					
					0.10 Hz band		0.15 Hz band		0.10 Hz band		0.15 Hz band		0.30 Hz band	
		TD [s]	Sigbin%	ROI	PD [s]	n [%]	PD [s]	n [%]	PD [s]	n [%]	PD [s]	n [%]	PD [s]	n [%]
6R1	29	− 0,5	23%	97	10,3	***29%***	6,7	***14%***	–	–	–	–	2,9	*99%*
18R1	28	2,2	36%	103	9,8	***47%***	6,9	***40%***	10,0	*22%*	6,5	*39%*	3,2	*26%*
3R1	26	1,7	24%	93	10,5	***4%***	6,5	***13%***	–	–	6,4	*15%*	3,6	*39%*
11R1	25	2,3	70%	93	9,8	***39%***	6,9	***54%***	9,9	*38%*	6,9	*48%*	–	*–*
24R1	25	1,5	26%	93	–	***–***	6,8	***2%***	–	–	5,9	*6%*	3,4	*63%*
16R4	24	0,8	31%	93	9,9	***11%***	6,4	***19%***	–	–	5,9	*1%*	3,0	*97%*
9R1	23	1,9	36%	93	9,9	***38%***	7,0	***33%***	–	–	–	*–*	3,0	*78%*
13R2	22	2,0	34%	103	11,7	***61%***	6,9	***33%***	–	–	–	*–*	2,8	*87%*
14R4	22	1,4	27%	93	9,9	***20%***	6,5	***10%***	–	–	–	–	3,2	*88%*
20R1	22	2,4	47%	93	9,6	***42%***	6,7	***24%***	–	–	6,0	*2%*	3,4	*65%*
Mean	**24,60**	**1,57**	**35,4%**		**10,16**	**32,3%**	**6,73**	**24%**	**9,95**	**30%**	**6,27**	**18%**	**3,17**	**71%**
SD	2,50	0,87	14,1%		0,64	17,9%	0,21	16%	0,07	11%	0,40	20%	0,26	26%

In addition, the results of phase-coupling between BOLD oscillations in brainstem (ROIs with the largest time delay are indicated) and RRI oscillations are shown.

Table 1 shows results of the phase-coupling analyses between BOLD and RRI oscillations in the 0.1–0.15 Hz band. Phase-coupling, studied in the brainstem ROIs (93, 97, 103) revealed either a positive time delay (pTD) or a negative time delay (nTD) in connection with the percentage of significant bins (sigbin%) (^24,25^). The former (pTD) is characteristic for neural BOLD components with RRI preceding BOLD oscillations, the latter (nTD) represents vascular BOLD components with BOLD preceding RRI oscillations. Only one participant (6R1) exhibited vascular BOLD components with origin in the baroreflex loop. In the majority of participants with pTDs between 0.8 and 2.4 s it can be assumed, that a pacemaker in brainstem was activated to manage the elevated anxiety level.

### Directed coupling in the HF (0.2–0.4 Hz) band

**Figure 1:**
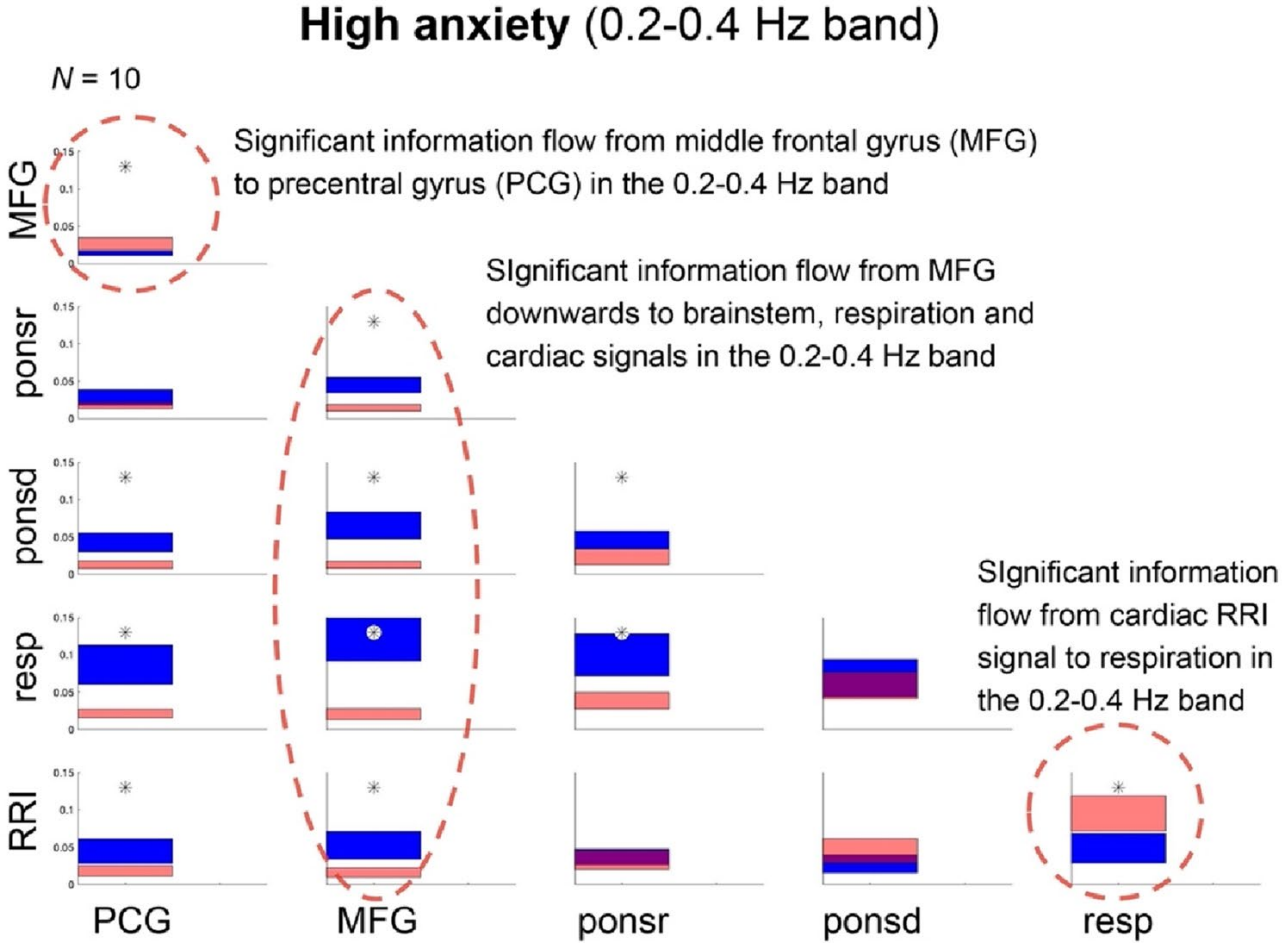
Directed coupling strengths for the high anxiety group in the 0.2–0.4 Hz frequency band. Each box shows the strength of coupling on the vertical scale. The height of bars is proportional to the mean error. The blue color shows the flow from the signal marked below the given column to the signal marked at the left, and the red color the flow from the signal marked at left to the signal marked below. Significant differences between couplings of inflow and outflow are marked by stars (*p* < 0.05). Note the highly-significant flow from the middle frontal gyrus (MFG, ROI 7) to the brainstem (ROIs 93 and 103), respiration and RRI (marked by a stippled ellipse), and the significant flow from the MFG to the precentral gyrus (PCG, ROI 1) and the significant flow from RRI to respiration (marked by a stippled circles).

The causal coupling analysis for the 0.2–0.4 Hz band revealed a strong information flow from middle frontal gyrus (MFG; ROI 7) to all other signals (Fig. 1). Most impressive was the coupling strength between BOLD in the MFG and the respiration signal (*p* < 0.001). In addition, a significant information flow from RRI to respiration signal was observed (*p* < 0.01). The precedence of slow RRI waves (relative to breathing waves) in the majority of subjects most likely indicate that RRI waves with oscillations at 0.16 Hz or 0.1 Hz drive breathing waves with a frequency ratio of 1:2 or 1:3 (2 or 3 breaths during one cardiac cycle). The direction of the flow is from RRI to breathing waves.

In Table 2 it can be noted the highly significant (*p* < 0.001) directed coupling between the BOLD signal from the MFG, respiration and BOLD oscillations in the brainstem. Such a significant flow was never observed before, neither in the LF or the 0.1–0.2 Hz bands. Therewith, it underlines both, the importance of respiratory oscillations around 0.3 Hz and the MFG as location for the downwards information flow.

**Table 2:** Significance of differences of flows from structures marked below the columns to the ones marked at the left of the rows.

ROI7	**98.7362**				
ROI93	5.9636	**0.0109**			
ROI103	**0.1241**	**0.0347**	**0.6344**		
resp	**0.0445**	**0.0199**	**0.1421**	29.4511	
RRI	**0.9221**	**0.3689**	29.1057	93.9989	**99.3602**
	ROI1	ROI7	ROI93	ROI103	Resp

The numbers show percentiles of the distributions obtained by the bootstrap approach (see^16,17^) for the high anxiety group in the 0.2–0.4 Hz band. Significant differences (marked by bold numbers) are those, which exceed the range of 95% of the random distribution of the differences obtained by the bootstrap method. Depending on the sign of the differences between in- and outflows, they are located below the 2.5 and above the 97.5 percentile.

### BOLD oscillations during fast respiration at ~ 0.32 Hz

High anxiety is accompanied by fast respiration at ~ 0.32 Hz, a strong downwards information flow from the MFG to the brainstem, and coupling between respiration and RRI signal in the 0.2–0.4 Hz band (Fig. 1). From intracranial EEG (iEEG) recordings in patients with medically intractable epilepsy it is known that nasal breathing at frequencies between ~ 0.16 and 0.32 Hz can reveal respiration-entrained limbic oscillations in the piriform (olfactory) cortex as well as in the amygdala and hippocampus^6,7^. One question is, whether fast respiration at ~ 0.32 Hz in healthy humans can also elicit fast oscillations in the limbic system.

**Figure 2:**
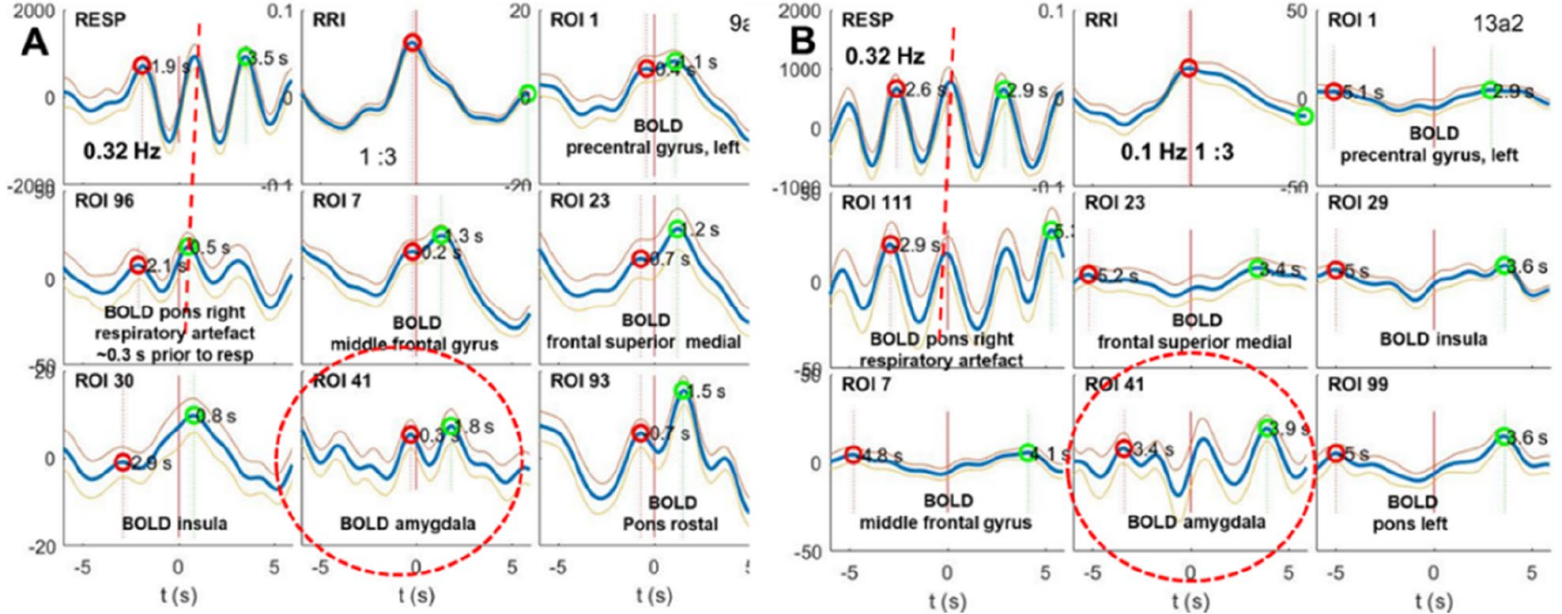
Examples of RRI peak (longest RR interval)-triggered breathing, RRI and BOLD waves (mean +  − SE) from two subjects (panel **A** and **B**) with prominent respiration at ~ 0.32 Hz. The locations of ROIs used for BOLD recordings are indicated. Note the BOLD artefact induced by vessel motion of the basilar artery in right pons/brainstem preceding respiration, measured from a chest belt (stippled vertical line). BOLD oscillations from amygdala are marked by stippled circles.

Examples of two typical subjects with high anxiety are displayed in Fig. 2A and B. The fast BOLD oscillations in the amygdala are centered at ~ 0.32 Hz and the slow oscillations in the PCG (ROI 1), MFG (ROI 7), medial frontal cortex (ROI 23) and brain stem (ROIs 93, 99) centered at ~ 0.16 Hz. While the former oscillations may be related to a downward information flow, the latter may have its origin in the brainstem pacemaker exhibiting an upward flow (Pfurtscheller et al.^17^). Both information flows are important, because they contribute to a power increase in the LF and HF band of the HRV, an important prerequisite for successful emotional regulation^26^. Note, these slow BOLD oscillations are not exactly time-locked to RRI oscillation but delayed.

### BOLD oscillations during respiration at ~ 0.16 Hz

**Figure 3:**
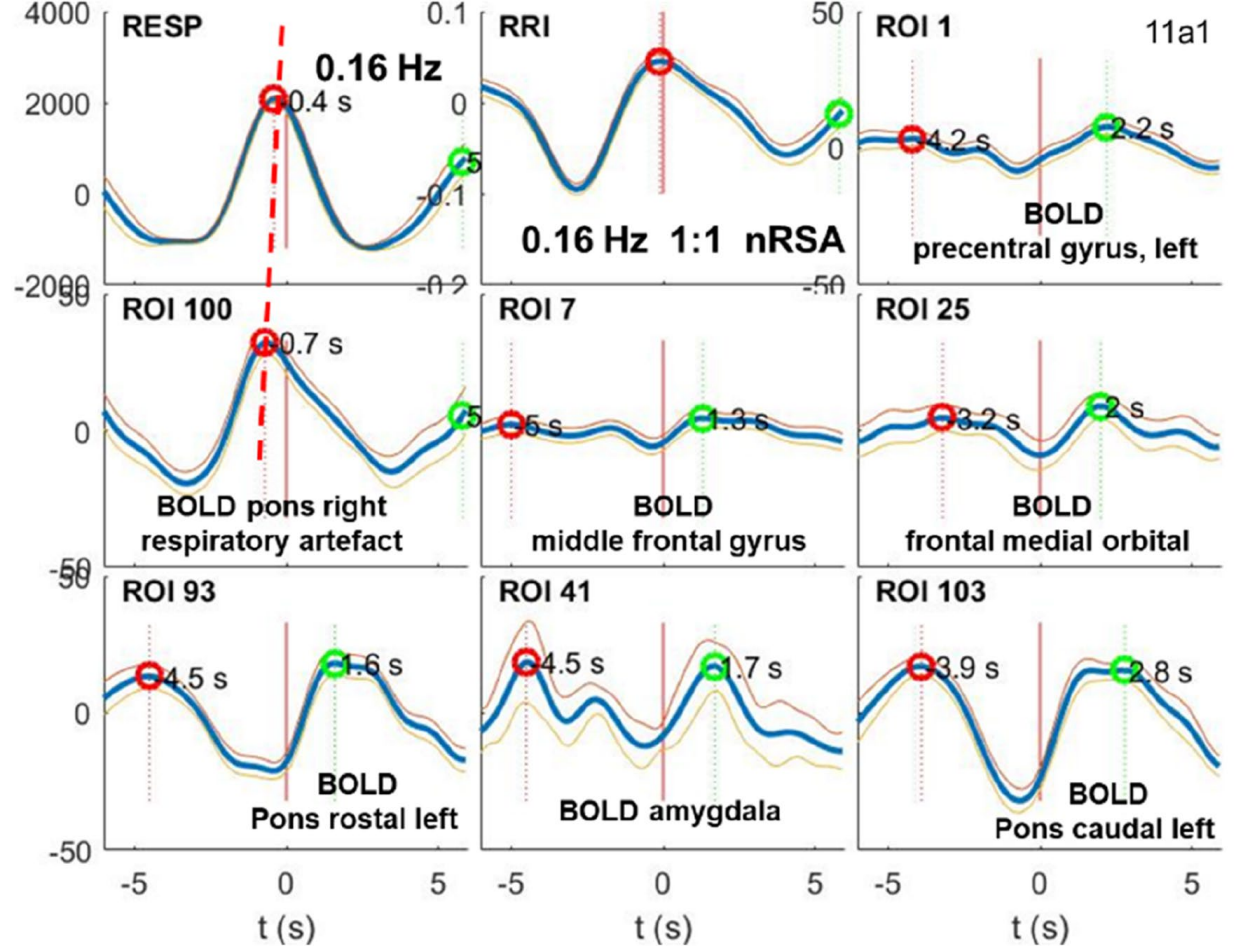
Examples of RRI peak triggered breathing and BOLD waves from a normal subject with high fMRI-related anxiety. For further explanation see Fig. 2. The data are from the first half of recording with dominant 0.16 Hz oscillations (in the second half dominated the 0.1 Hz oscillations were prevailing) and precise waves with period s of 6.2 s in amygdala.

In addition to the dominating breathing waves at 0.32 Hz during high anxiety, one subject exhibited 0.16 Hz and 0.1 Hz oscillations, the former in the first half of recording and the latter in the second half. RRI peak-triggered averaged waves from the first half are displayed in Fig. 3. Most impressive are the coherent breathing and RRI waves with a period of ~ 6.3 s (corresponds to 0.16 Hz) and the distinct BOLD oscillations in the cortex, amygdala and brainstem, delayed from the breathing and RRI waves by ~ 2–3 s. This subject exhibited a significant upwards information flow from the brainstem to the cortex in the 0.1–0.2 Hz band (Pfurtscheller et al. 2022). Remarkable in Fig. 3 are the smooth slow BOLD waves in the precentral cortex and brainstem and the rippled BOLD wave in the amygdala.

## Discussion

The wave-by-wave analysis revealed in all subjects, except one, a duration of breathing waves with 3.2 s +  − 0.26 (mean ± SD), corresponding to a breathing frequency at ~ 0.31 Hz^12^. Two findings are of particular interest, (i) the dominant fast respiration at ~ 0.32 Hz and the top-down flow (Fig. 1) and (ii) the bottom-up flow at ~ 0.16 Hz (Fig. 3).

These two frequencies correspond to two preferred breathing frequencies of the binary hierarchy model^11,27^. The principle of this model is, that the frequency of body oscillations (respiration, heart rate) can be predicted from brain oscillations (EEG alpha and beta rhythms). The model includes three preferred breathing frequencies at 0.08 Hz, 0.16 Hz, and 0.32 Hz, exhibiting a doubling-halving frequency relationship. In this hierarchy especially the breathing frequencies at 0.32 Hz and 0.16 Hz are of interest. This model shows that synchronization processes exist in various ways and varying degrees between neural activity in peripheral neural clusters, such as the heart and stomach and brain rhythms, maintaining a general steady state during waking consciousness^28^.

### Fast BOLD oscillations of neural and non-neural origin

The recording of BOLD oscillations with frequencies in the HF (0.2–0.4 Hz) band in the scanner, especially from amygdala (ROI 41), is a great challenge. The sampling rate of 1.15 Hz (corresponds to TR = 0.871 s) allows to record correctly sinusoidal frequency components <  = 0.58 Hz. The problem is the respiratory BOLD artefact due to vessel motion (vascular BOLD), whereby areas with large blood vessels and good perfusion are affected. Such regions are localized e.g., close to the cingulum supplied by the middle cerebral artery^29^ and the pons with the basilar artery. Interestingly, not only slow respiratory BOLD artefacts in pons (ROI 96, 98,100) are dominant about 0.3 s prior the respiratory signal measured with chest belt (examples see Fig. 3 in^30^) but also fast respiratory BOLD artefacts as documented in Fig. 2A and B). This short delay between respiratory signal and vascular BOLD signal is indicated by the connection of equivalent peaks by a stippled line. The vascular BOLD signal is considered to result from a respiratory modulation of sympathoexcitatory neurons in the rostroventrolateral reticular nucleus^31^ causing maximal vasodilatation (maximum of the BOLD signal) immediately preceding the start of expiration and vasoconstriction (minimum of the BOLD signal) just before the start of inspiration^30^. The observation indicates that the BOLD artefact in the HF band can be reconstructed correctly. This, provides evidence that neural BOLD oscillations in the HF band can be studied. We speculate therefore, that fast BOLD oscillations are of neural origin and dominant in the amygdala. They are delayed relatively to the respiratory BOLD artefact through the neuro-BOLD coupling time of about 2–3 s^13,32,33^. The problem is that in the case of wave periods shorter than 5 s, no clear separation of vascular and neural BOLD signals is possible. Therefore, the superposition of both BOLD signals can result in a truncation of the neural BOLD response.

### Breathing oscillations at ~ 0.32 Hz and top-down flow

A novel finding is the saliency of fast respiration at ~ 0.32 Hz during high anxiety. Fast respiration centered around the preferred breathing frequency at 0.32 Hz covers a broad range from 0.25 to 0.39 Hz^11^. Interestingly, already normal subjects while sitting in a quiet room and breathing quietly can be separated into two groups. From these, the group with higher anxiety revealed a breathing frequency of 0.28 Hz^34^. Both, Zelano et al.^6^ and Herrero et al.^7^ studied intracranial EEG (iEEG) in patients with medically intractable epilepsy. Zelano et al.^6^ controlled nasal breathing by a pneumotachometer positioned in front of the nostrils and measured mouth breathing with a chest belt. In the patients studied, a breathing rate between 0.24 and 0.36 Hz was dominant. This main finding provides strong evidence for nasal respiratory-entrained local field potential activity in human piriform cortex, amygdala, and hippocampus, whereby these fast oscillations in PFC are diminished by mouth breathing. It is assumed that such patients have a burden of anxiety, whereby the level of anxiety is unknown. A similar breathing rate around 0.31 Hz during high fMRI-related anxiety was reported in healthy subjects^12^.

An interesting point is the relationship between fast nasal breathing in patients and fast breathing of healthy people in the scanner during high anxiety. In the former case it is very likely that patients with medically intractable epilepsy are not free from anxiety, in the latter case the fast breathing might be one strategy or mechanism to manage high anxiety processing. From directed coupling measures there is strong evidence, that a downwards flow from the cortex to the brainstem in the 0.2–0.4 Hz band can be a marker for nasal breathing.

### Breathing oscillations at ~ 0.16 Hz with bottom-up and top-down information flow

**Figure 4:**
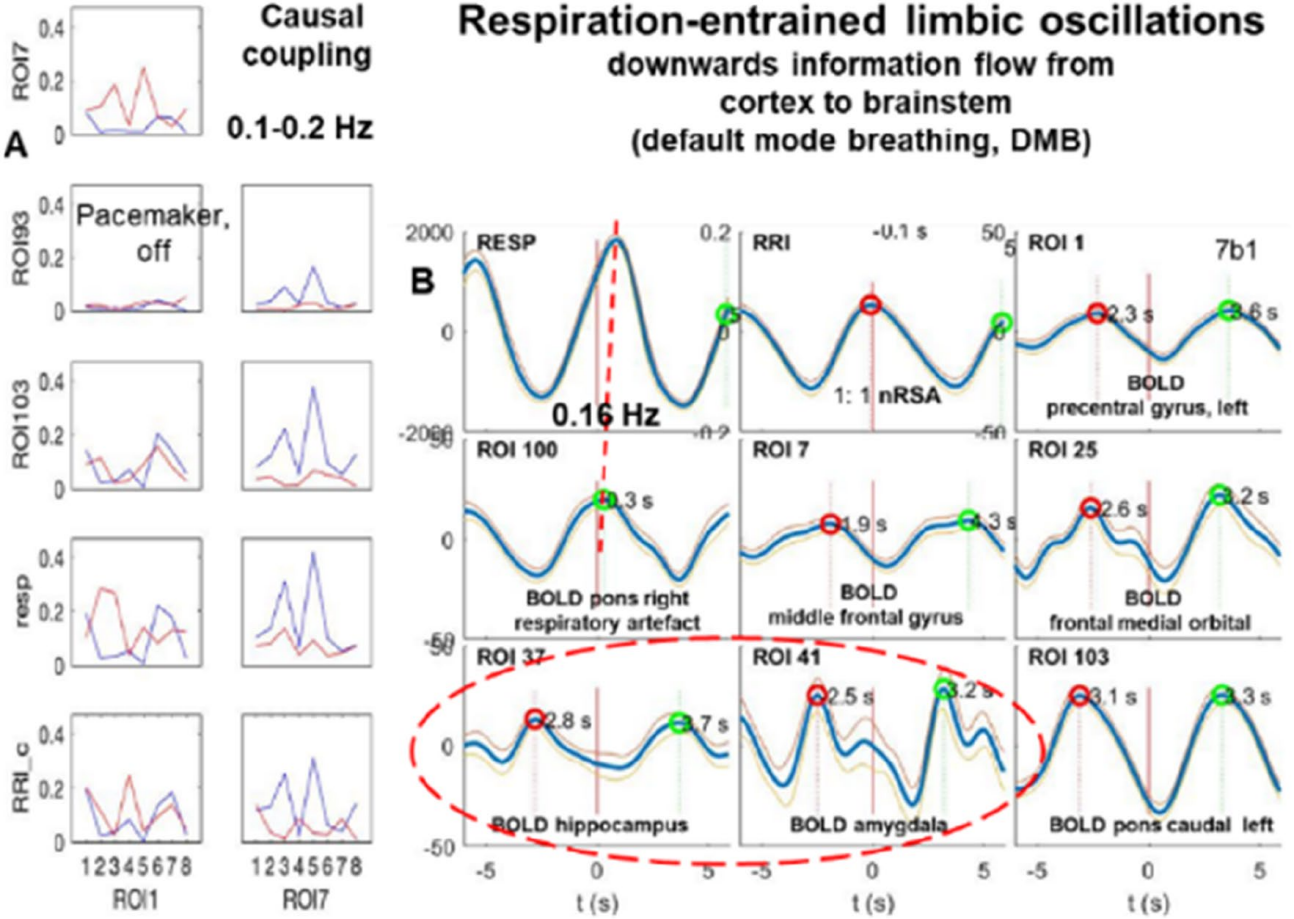
(**A**) Results of DTF analyses. In each box ffDTF as a function of consecutive eight time windows of 40 s length is shown. Blue lines: outflow from the structure marked below the box to the structure marked at the left, red lines: flow from structure marked at the left to this marked below (Modified from^17^). (**B**) Examples of RRI peak triggered breathing and BOLD waves from a normal subject with low fMRI-related anxiety. For further explanation see Fig. 2. Note, the similarity of BOLD oscillation at ~ 0.16 Hz in limbic system (marked by stippled ellipses), prefrontal cortex (ROIs 1and 7) and brainstem. (ROI 103).

In contrast to BOLD oscillations of neural origin at frequencies around 0.32 Hz and their possible overlap with a respiratory BOLD artefact, slow neural BOLD signals at 0.16 Hz are more easily to interpret, especially if they appear coherent with respiration (Fig. 3). For the interpretation of the directed coupling data from 10 subjects shown in Fig, 1, it may be helpful to present one example with low anxiety and top-down flow from MFG in the 0.1–0.2 Hz band (Fig. 4A) and averaged BOLD waves (Fig. 4B). Remarkably are in this case the coherent BOLD oscillations at 0.16 Hz, which were found in the PFC, MFG, brainstem and limbic system (amygdala and hippocampus).

Noteworthy is the great similarity of BOLD waves in the amygdala (ROI 41) in different subjects (Figs. 3 and 4). Both figures display very similar patterns with coherent beathing and RRI waves, coherent BOLD waves in the left amygdala and left caudal pons structure (ROI 103), but opposed information flows. In case of Fig. 3, the information flow is bottom-up from the brainstem to the PFC and in case of Fig. 4, it is top-down from the PFC to the brainstem. The latter case of downwards directed coupling is a good example of respiration-entrained brain oscillations, and the former case, upwards directed coupling, can be seen as example of pacemaker-entrained brain oscillations. This information flow arrives from a pacemaker-like rhythmic source localized in reticular structures of the brainstem^13,35,36^. It generates rhythmic activity basically around 0.16 Hz and respiratory oscillations at the same frequency (ratio 1:1) or the double frequency (ratio 1:2). Note, these upwards coupling may suggest another possible pathway from the preBötzinger complex in the brainstem to the locus coeruleus and, moreover, via the medial thalamus and the olfactory bulb to the PFC and the limbic system^37,38^.

Table 1 exhibits slow RRI waves (PD = 6.7 s +  − 0.21) of various appearance between 2% and 54% in all subjects with high anxiety. It is not clear, whether they are pacemaker- entrained or respiration- entrained. Motor functions such as eye or finger tracking movements can be entrained by respiration(^39,40^ indicating that respiration can modulate and even entrain central neural rhythms. Respiration-entrained brain rhythms are global but often overlooked and have been preferably studied in the rodent brain^2,4,41,42^. There is a great scientific interest in such an entrainment in humans, because the respiratory rhythm can have a direct influence on cortical oscillations such as EEG theta and gamma oscillation^43^. Further research on this issue is needed.

Recently, data from 28 healthy volunteers during a steady contraction task of the forearm have been recorded in a 275 channel whole head magnetoencephalography (MEG) study with visually monitored deep nasal breathing^44^. In this study, the corticomuscular coherence was synchronized with respiration showing a maximum over the sensorimotor cortex. Noteworthy, the dominant breathing rate was 0.14 Hz, which is close to the preferred breathing frequency of 0.16 Hz of the binary hierarchy model^11^.

## Conclusions

During anxiety, three body oscillations with frequencies at around 0.32 Hz, 0.16 Hz and 0.1 Hz are of interest. The former two exhibit a doubling- halving frequency relationship and are predicted by the binary hierarchy model^11^. The latter frequency does not belong to the binary hierarchy, but may play an important role in combination with 0.16 Hz. Rassler et al.^12,45^ reported two center frequencies in coherent breathing and cardiac RRI waves, one at ~ 0.16 Hz and another at ~ 0.1 Hz. Preferred breathing oscillations at 0.32 Hz with a period of 3.13 s are very frequent during high anxiety processing and exhibit often doubling during one RRI wave at 0.16 Hz and triplication during one RRI waves at 0.1 Hz.

Here we suggest the hypothesis that nasal breathing is responsible for relatively slow breathing at 0.16 Hz during low anxiety. Slow breathing appears to represent a default mode in a state of alert wakefulness when nasal breathing dominates. Experience from everyday observations suggests that awake healthy persons typically prefer nasal breathing at rest. Studies on airway flow through the oral and nasal breathing routes confirmed that more than 80% of healthy subjects breathe predominantly or even exclusively through the nose at rest^46,47^.

In contrast, relatively fast breathing at 0.32 Hz can be observed during high anxiety. Whether breathing at 0.32 Hz is also associated with nasal breathing is not completely clear, although causal coupling revealed a significant information flow from prefrontal cortex to centers in the brainstem in the band 0.2–0.4 Hz. In contrast to this highly significant downwards information flow, an ascending flow in the band 0.1–0.2 Hz was reported by Pfurtscheller et al.^17^. In this latter case the source of slow rhythmic activity is very likely a pacemaker in the brainstem. Note, that pacemaker induced activity from the brainstem operates either at a center frequency of 0.16 Hz or 0.1 Hz and modulates breathing oscillations either in a 1:1 (coherent breathing and RRI waves), 1:2 or 1:3 (two or three breaths at one cardiac wave) manner.

One core function of the brain is to efficiently coordinate and regulate energetic requirements of the body. Examples are the pacemaker-entrained ~ 0.16-Hz oscillations and the preferred breathing oscillations at ~ 0.16 Hz of the binary hierarchy model of Klimesch^11^. Of interest are also adaptively altering physiological activities of the body (e.g. respiration and heart rate) that respond to environmental changes (e.g. changes due to claustrophobia during scanning) known as allostasis^48–50^. Possible examples of allostasis are the respiration-entrained 0.16-Hz oscillations during low anxiety, the pacemaker-entrained 0.16 Hz oscillations, and the 0.32 Hz respiration oscillations during high anxiety^16,17^. The importance of respiration as integral rhythm of the brain’s neural activity is highlighted in two recently published papers from Northoff’s group^51,52^. Respiration is less a confounder variable in fMRI data, but more an intrinsic part of the brain's entrainment and synchronization processes.

## Data Availability

The datasets generated during and/or analyzed during the current study are available from the corresponding author on reasonable request.
